# Paralysie périodique hypokaliemique thyrotoxique chez un Noir africain à Abidjan (Côte d’Ivoire)

**DOI:** 10.48327/mtsi.v4i4.2024.543

**Published:** 2024-11-15

**Authors:** Claude Valéry Cédric Aka KADJO, Ségla Achi Cédric AGBOPANZO, Fiacre Delors OFFOUMOU, Arlette Désirée AKA

**Affiliations:** Service de neurologie, Centre hospitalier universitaire (CHU) de Cocody, Université Félix Houphouët Boigny, UFR des sciences médicales d’Abidjan, Cote d’Ivoire

**Keywords:** Paralysie hypokaliémique, Thyrotoxique, Africain noir, Côte d’Ivoire, Afrique subsaharienne, Hypokalemic paralysis, Thyrotoxic, Black African, Côte d’Ivoire, Sub-Saharan Africa

## Abstract

**Introduction:**

La paralysie périodique hypokaliémique thyréotoxique (PPHT) est une forme sporadique de la paralysie périodique hypokaliémique (PPH). Il s'agit d'une urgence diagnostique et thérapeutique rarement décrite chez le sujet noir. Nous rapportons un cas chez un sujet noir ivoirien.

**Cas clinique:**

M. NK âgé de 37 ans, a été hospitalisé pour un déficit moteur des quatre membres d'installation rapidement progressive. Le patient a une hyperthyroïdie traitée par néomercazole. Son histoire révèle un premier épisode régressif en 30 mn puis une récidive plus marquée 4 mois plus tard dans un contexte d'arrêt thérapeutique. L'examen clinique a noté une tétraparésie flasque affectant principalement les membres inférieurs. Le bilan biologique a retrouvé une hypokaliémie à 2,6 mEq/L, la TSH ultrasensible était basse (inférieure à 0,005 µl/ml) avec une élévation des T3 et T4 respectivement à 24,42 µl/ml et 79,68 µl/ml. Nous avons retenu le diagnostic d'une PPHT. L’évolution clinique a été satisfaisante après la correction de la kaliémie puis une réadaptation du traitement de l'hyperthyroïdie.

**Discussion:**

La PPHT est fréquente chez le sujet jeune asiatique et rare chez le sujet africain noir. La durée des épisodes paralysants varie de 1 à 72 heures avec une moyenne de près de 24 heures. La paralysie des muscles respiratoires, potentiellement grave, est rare. Cependant, l'arrêt thérapeutique chez les patients déjà sous traitement peut être considéré comme favorisant la survenue de paralysie.

**Conclusion:**

L’évolution favorable de la paralysie après correction de la kaliémie est avérée. Notre observation souligne l'intérêt du maintien d'un bon fonctionnement thyroïdien chez les patients suivis pour une hyperthyroïdie.

## Abstract (en)

**Introduction:**

Thyrotoxic hypokalemic periodic paralysis (THPP) is a sporadic form of hypokalemic periodic paralysis (HPP). It is a diagnostic and therapeutic emergency rarely described in the black population. We report a case in a black subject from Côte d’Ivoire.

**Clinical case:**

Mr. NK, 37 years old, was admitted to hospital with rapidly progressive motor deficit in all four limbs. The patient had hyperthyroidism, which was treated with neomercazole. His medical history revealed an initial episode that resolved within 30 minutes, followed by a more severe recurrence 4 months later, associated with discontinuation of treatment. Clinical examination revealed flaccid tetraparesis mainly affecting the lower limbs. Biological tests showed hypokalemia of 2.6 mEq/L, ultrasensitive TSH was low (less than 0.005 µL/mL) with T3 and T4 elevations of 24.42 µL/mL and 79.68 µL/mL respectively. We have retained the diagnosis of THPP. The clinical course was satisfactory after correction of the kalemia and readjustment of the hyperthyroidism treatment.

**Discussion:**

THPP is common in young Asians and rare in black Africans. The duration of paralysis varies from 1 to 72 hours, with an average of almost 24 hours. Respiratory paralysis, which is potentially serious, is rare. However, discontinuation of treatment in patients already on the drug may predispose to paralysis.

**Conclusion:**

The favorable evolution of paralysis after correction of kalemia was confirmed. Our observation highlights the importance of maintaining good thyroid function in patients monitored for hyperthyroidism.

## Introduction

La paralysie périodique hypokaliémique thyréotoxique (PPHT) est une forme sporadique de la paralysie périodique hypokaliémique. La PPHT est une complication rare et potentiellement grave de l'hyperthyroïdie [9]. Il s'agit d'une pathologie rarissime, plus fréquente chez le sujet masculin asiatique [9], caractérisée par des épisodes de paralysie ou de faiblesse musculaire pouvant toucher les muscles respiratoires et donc présenter un risque létal potentiel [3]. Il s'agit d'une urgence diagnostique et thérapeutique rarement décrite chez le sujet noir. Nous rapportons un cas chez un sujet noir ivoirien.

## Cas clinique

M. NK ivoirien d'origine âgé de 37 ans, a été hospitalisé aux urgences médicales pour une tétraparésie d'installation rapidement progressive constatée à son réveil le 30 mars 2024. La veille, le patient aurait présenté des myalgies diffuses. Le patient a comme antécédent une hyperthyroïdie diagnostiquée en novembre 2023, pour laquelle un traitement par néomercazole a été instauré. L'histoire du patient révèle un épisode de tétraparésie sans caractère ascendant en novembre 2023 ayant duré environ 30 min, et suivi d'une régression spontanée progressive du déficit moteur dans un contexte apyrétique. Le 29 mars 2024, le patient a présenté un déficit moteur des quatre membres constaté au réveil et d'aggravation progressive. Le patient a indiqué un arrêt thérapeutique d'une semaine avant le début des symptômes. Ce déficit moteur a été précédé la veille par des myalgies diffuses. L'examen à son admission en hospitalisation a noté une tétraparésie flasque avec une force motrice à 3/5 affectant surtout les membres inférieurs. Le bilan biologique a retrouvé une hypokaliémie à 2,6 mEq/l. La natrémie et la chlorémie ainsi que les fonctions rénales étaient normales. La TSH ultra sensible était basse (inférieure à 0,005 µl/ml) avec une élévation des T3 et T4 respectivement à 24,42 µl/ml et 79,68 µl/ml. Nous avons retenu le diagnostic d'une paralysie périodique hypokaliémique thyréotoxique. La prise en charge a consisté en une perfusion de 3 g de chlorure de potassium. L’évolution après 24 heures a été marquée sur le plan clinique par une amélioration de la force motrice. Le bilan biologique a retrouvé une kaliémie normale à 3,9 mEq/l. Le patient est sorti à 48h après, devant une évolution clinique satisfaisante avec suivi en endocrinologie.

## Discussion

La paralysie hypokaliémique est une forme rare de manifestation de l'hypokaliémie. Elle peut se manifester selon diverses modalités, notamment la forme thyrotoxique associant une hyperthyroïdie à la paralysie périodique. Cette forme est plus fréquente chez le sujet asiatique de sexe masculin. Une autre présentation est la forme associant une hypernatrémie; la présence d'une tumeur cérébrale ou d'une tuberculose est souvent mise en évidence. La paralysie périodique peut aussi être familiale, sans hyperthyroïdie ou hypernatrémie associée. Cette situation est la plus retrouvée en Europe. Enfin, il existe une paralysie périodique sporadique, sans hyperthyroïdie, sans hypernatrémie et sans aucune notion familiale [7, 9].

La PPHT n'est pas causée par des anomalies génétiques, mais une association avec CACNA1S 5’UTR et des polymorphismes nucléotidiques introniques a été suggérée, sans toutefois être confirmée. Un variant pathogène dans un canal potassique (Kir) à rectification interne (codé par le gène *KCNJ18)* a été identifié chez environ un tiers des personnes atteintes dans une série [10]. La PPHT est une affection rare chez le sujet de race blanche [5]. Les cas décrits chez les sujets noirs proviennent essentiellement d’Amérique du Nord [4]. En Afrique noire, quelques-uns sont retrouvés dans la littérature [1, 2, 8]. La PPHT est caractérisée par une paralysie d'intensité variable, de durée variant de 1 à 72 heures avec une moyenne de près de 24 heures [10]. La paralysie est purement motrice, sans trouble sensitif ou végétatif, souvent déclenchée par un repas riche en glucides, un épisode infectieux ou une activité physique [6]. Cependant, l'arrêt thérapeutique chez les patients déjà sous traitement peut être considéré comme favorisant la survenue de paralysie, comme l'a montré l’étude de Sow [8].

La gravité de la PPHT réside dans l'atteinte des muscles respiratoire qui reste cependant rare [4]. La prise en charge globale passe par la supplémentation potassique et la correction de l'hyperthyroïdie. Cependant, dans les formes sévères, l'apport potassique doit se faire par voie veineuse lente afin de prévenir la survenue de trouble du rythme et la paralysie des muscles respiratoires [9]. Le traitement préventif des paralysies repose sur le maintien de la fonction thyroïdienne normale [10].

## Conclusion

La paralysie périodique hypokaliémie thyrotoxique est rare en Afrique noire. Il s'agit d'une urgence thérapeutique du fait du risque d'atteinte respiratoire et de survenue de trouble du rythme cardiaque. L’évolution favorable de la paralysie après correction de la kaliémie est démontrée. Notre observation souligne l'intérêt du maintien d'un bon fonctionnement thyroïdien chez les patients suivis pour une hyperthyroïdie.

## Contributions des auteurs et de l'autrice

KADJO Claude Valéry Cédric Aka : conception du rapport de cas, prise en charge diagnostique et thérapeutique du patient, rédaction, révision et validation manuscrit.

AGBO-PANZO Ségla Achi Cédric : prise en charge diagnostique et thérapeutique du patient, rédaction, révision et validation manuscrit OFFOUMOU Fiacre Delors : rédaction, révision et validation manuscrit

AKA Arlette Désirée : rédaction, révision et validation manuscrit

## Remerciements

Amon-Tanoh Muriel, Kouassi Dihoiba, Toa Goury Axel, Baugnan Davide Parfaite, Tanoh Christian Abel.

## Consentement éclairé

Nous avons obtenu le consentement oral du patient.

## Conflits d'intérêt et principes éthiques

Les auteurs et l'autrice ne déclarent aucun conflit d'intérêt.
