# Supplementary material for: Thyrotoxic hypokalemic periodic paralysis in a black African man in Abidjan (Côte d’Ivoire)
Source: Med Trop Sante Int. 2024 Nov 15;4(4):mtsi.v4i4.2024.543. [Article in French] doi: 10.48327/mtsi.v4i4.2024.543 (PMC11892388; doi:10.48327/mtsi.v4i4.2024.543)
Supplement: Related texts

# Research article: Thyrotoxic hypokalemic periodic paralysis in a black African man in Abidjan (Côte d’Ivoire)

## Introduction

Thyrotoxic hypokalemic periodic paralysis (THPP) is a sporadic form of hypokalemic periodic paralysis. THPP is a rare and potentially serious complication of hyperthyroidism [9]. It is a rare condition, more common in Asian males [9], characterised by episodes of paralysis or muscle weakness that can affect the respiratory muscles and is therefore potentially fatal [3]. It is a diagnostic and therapeutic emergency rarely described in black subjects. We report a case in a black subject from Côte d’Ivoire.

## Clinical case

Mr. NK from Côte d’Ivoire, aged 37 years, was admitted to a medical emergency department on 30 March 2024 with rapidly progressive tetraparesis on awakening. The patient had reportedly presented with diffuse myalgia the day before. The patient had a history of hyperthyroidism, diagnosed in November 2023, for which treatment with neomercazole had been initiated. The patient's medical history showed an episode of tetraparesis without ascending character in November 2023, lasting approximately 30 minutes, followed by a progressive spontaneous regression of the motor deficit in an apyretic context. On 29 March 2024, the patient presented with a motor deficit in all four limbs, which was noted on awakening and progressively worsened. The patient reported that he had stopped taking his medication one week before the onset of symptoms. This motor deficit was preceded by diffuse myalgia the day before. On admission to hospital, the patient was found to have flaccid tetraparesis with a motor strength of 3/5, mainly affecting the lower limbs. Biological tests showed hypokalemia of 2.6 mEq/L. Natriuresis and chloremia were normal, as were renal functions. The ultrasensitive TSH was low (less than 0.005 µL/mL) with T3 and T4 elevations of 24.42 µL/mL and 79.68 µL/mL respectively. The diagnosis was hypokalemic thyrotoxic periodic paralysis. Treatment consisted of an infusion of 3 g of potassium chloride. The clinical course after 24 hours was characterised by improvement in motor strength. Biological tests showed a normal potassium level of 3.9 mEq/L. The patient was discharged after 48 hours with a satisfactory clinical course and endocrinological follow-up.

## Discussion

Hypokalemic paralysis is a rare manifestation of hypokalaemia. It can manifest in a variety of ways, in particular the thyrotoxic form, which combines hyperthyroidism with periodic paralysis. This form is more common in Asian males. Another form is associated with hypernatremia; the presence of a brain tumour or tuberculosis is often detected. Periodic paralysis may also be familial, without associated hyperthyroidism or hypernatremia. This situation is the most common in Europe. Finally, there is sporadic periodic paralysis, with no hyperthyroidism, no hypernatremia and no family history [7, 9]. THPP is not caused by genetic abnormalities, but an association with CACNA1S 5’UTR and intronic nucleotide polymorphisms has been suggested but not confirmed. A pathogenic variant in an internally rectifying potassium (Kir) channel (encoded by the KCNJ18 gene) has been identified in approximately one-third of affected individuals in one series [10].

THPP is a rare condition in Caucasians [5]. The cases described in black subjects come mainly from North America [4]. In black Africa, a few cases have been reported in the literature [1, 2, 8]. THPP is characterized by paralysis of variable intensity, lasting from 1 to 72 hours, with an average of almost 24 hours [10]. The paralysis is purely motor, with no sensory or vegetative disturbance, and is often triggered by a meal rich in carbohydrates, an infectious episode or physical activity [6]. However, discontinuation of treatment in patients already on it may be considered to favor the onset of paralysis, as shown in Sow's study [8].

The seriousness of THPP lies in damage to the respiratory muscles, although this remains rare [4]. Overall management involves potassium supplementation and correction of hyperthyroidism. However, in severe forms, potassium supplementation should be administered by slow venous route in order to prevent the onset of rhythm disorders and respiratory muscle paralysis [9]. Treatment to prevent paralysis is based on maintaining normal thyroid function [10].

## Conclusion

Thyrotoxic hypokalemic periodic paralysis is rare in sub-Saharan Africa. It is a therapeutic emergency because of the risk of respiratory and cardiac arrhythmias. There is evidence of a favourable outcome of the paralysis after correction of the hypokalemia. Our observation highlights the importance of maintaining good thyroid function in patients treated for hyperthyroidism.

## Authors’ contributions

KADJO Claude Valéry Cédric Aka: design of the case report, diagnostic and therapeutic management of the patient, drafting, revision and validation of the manuscript.

AGBO-PANZO Ségla Achi Cédric: diagnostic and therapeutic management of the patient, drafting, revision and validation of the manuscript.

OFFOUMOU Fiacre Delors: drafting, revision and validation of the manuscript.

AKA Arlette Désirée: drafting, revision and validation of the manuscript.

## Acknowledgments

Amon-Tanoh Muriel, Kouassi Dihoiba, Toa Goury Axel, Baugnan Davide Parfaite, Tanoh Christian Abel.

## Informed consent

Oral consent was obtained from the patient.

## Conflicts of interest and ethics

The authors declare no competing interests.
